# Mind Reading

**Published:** 1890-07

**Authors:** 


### MIND READING.

The most remarkable of mind reading exploits, known as the wire test, was given by J. Randolph Brown, in the presence of a large party of Congressmen and others. An insulated copper wire was used. One end of it was held by Brown across his forehead. The other end wa taken possession of by a gentleman selected from the party and a stranger to Brown. At a signal, this gentleman placed the end of the wire against his forehead. He opened his watch and looked at the number engraved upon it. Brown, at the other end of the wire, and with his eyes blindfolded, wrote the number, figure by figure, upon a blackboard. The test was performed under such conditions as to make fraud or trickery impossible. The gentleman who opened his watch frankly admitted that he did not know what the number was until then. The figures were small. He made a mistake in one figure, thinking a six a five. The mental telegraph was true to the blunder. The figures which Brown had traced upon the blackboard were exactly as the gentleman thought he saw them on his watch. There was absolutely no possibility of communication between the two men except by the wire. It was a clear case of mind telegraphing to mind. Brown has been experimenting on distances. Ex-Governor James Pollock, of Pennsylvania, who died recently, held the wire at Wilmington not long ago, while Brown, at the Philadelphia end, twenty-eight miles away, successfully wrote numbers Pollock fixed upon his mind. This wire feat of Brown's is far in advance of any thing which has hitherto been performed in the way of mind reading.

